# Risks of dairy derived excipients in medications for lactose intolerant and cow milk protein allergic patients

**DOI:** 10.1038/s41598-024-66380-8

**Published:** 2024-07-07

**Authors:** Alexandra Figueiredo, Maria Deolinda Auxtero, Maria Santo, Andreia Casimiro, Isabel Margarida Costa

**Affiliations:** 1 Instituto Universitário Egas Moniz (IUEM), Campus Universitário - Quinta da Granja, 2829-511 Monte de Caparica, Portugal; 2 PharmSci Lab/ Centro de Investigação Interdisciplinar Egas Moniz (CiiEM), Campus Universitário - Quinta da Granja, 2829-511 Monte de Caparica, Portugal

**Keywords:** Pharmaceutical excipients, Lactose intolerance, Cow milk protein allergy, Dairy-derived ingredients, Cross-contamination risk, Health care, Risk factors

## Abstract

The use of lactose and cow milk protein (CMP) as potential allergens in pharmaceuticals and their ability to cause allergic reactions remains a significant concern in medicine. Lactose, a common pharmaceutical excipient due to its inert, inexpensive, and stable properties, is found in many prescription-only and over-the-counter medications. However, despite their widespread use, individuals with lactose intolerance (LI) or cow milk protein allergy (CMPA) may experience adverse reactions to these excipients. This study investigated the prevalence of lactose and other dairy-derived ingredients in pharmaceuticals marketed in Portugal. Using the Summary of Product Characteristics (SmPC) from the INFOMED database, various medications, including analgesics, antipyretics, non-steroidal anti-inflammatory drugs (NSAIDs), and antiasthmatics, were analyzed. Results showed a high prevalence of dairy-derived excipients, particularly in antiasthmatic drugs (62.6%) and NSAIDs (39%). Although CMP are not explicitly mentioned in SmPCs, the presence of lactose as an ingredient poses a risk of cross-contamination. The findings emphasize the need for healthcare professionals to be aware of potential allergens in medications and the importance of developing lactose-free alternatives to ensure the safety of patients with LI and CMPA. Further research is required to assess the safety and implications of lactose in medicines for these populations.

## Introduction

Currently, the use of lactose and cow milk protein (CMP) in medicine, and their potential to trigger allergic reactions, remains a topic of significant interest and concern in the medical field. Lactose is the predominant sugar in milk and is unique to dairy products. Owing to its characteristics—being inert, inexpensive, non-toxic, water-soluble and chemically stable^1,2^. This substance is one of the most widely used excipients in the pharmaceutical industry. It is found in 20% of prescription-only medicines and 65% of over-the-counter medicines^2–4^. Its role as an excipient varies, and it is a common constituent in several pharmaceutical dosage forms, such as tablets, capsules, dry powder inhalers, lyophilized products, sugar-coating solutions, and some liquid preparations^3,5^. Tablets represent the most common dosage form in which lactose is a diluent or filler. Lactose is often used as a carrier in dry powder inhaler (DPI) formulations^6^.

For absorption, lactose must be hydrolyzed by the lactase enzyme present on the surface of the mucosa of the small intestine^7^. In individuals with lactase deficiency, lactose is not properly digested and cannot be absorbed, remaining intact in the colon where bacteria convert it into gases and short-chain fatty acids. Fermentation products and unfermented lactose can cause gastrointestinal symptoms that make up the clinical picture of lactose intolerance (LI)^6,8,9^. Despite the widespread use of lactose, only 30% of the world’s adult population retains the ability to digest lactose effectively. High rates of lactase activity retention have been observed in Northern European populations (Scandinavia, Netherlands, United Kingdom), reaching up to 90%. Conversely, lactase deficiency is prevalent in 70–100% of African, South American and Asian populations^4^. For people with LI, lactose in medications can lead to digestive issues. Nevertheless, it is important for both healthcare providers and patients to know the amount of lactose that can cause symptoms in those who are lactose intolerant. Research indicates that it usually takes about 10 g of lactose per day to cause noticeable symptoms in most individuals who lack the enzyme lactase^10^. Since most formulations contain less than 1 g of lactose per dosage unit (capsule, tablet)^1,6^, medication is unlikely to cause significant lactose-related symptoms in lactase-deficient patients. However, some people with LI report experiencing symptoms even with small amounts of lactose, such as 100 to 200 mg, highlighting the significant variability in individual tolerance levels^1,2,11^. This variability can depend on numerous factors including genetic background, the specific composition of the gut microbiome, and the presence of other gastrointestinal conditions^12^. Moreover, the cumulative effect of multiple medications containing lactose should not be overlooked.

In addition to LI, the consumption of dairy products is also associated with other diseases, such as cow milk protein allergy (CMPA). CMP is the most common allergen in the pediatric population. Although the exact prevalence is challenging to monitor, it is estimated that approximately 0.5–6% of infants in developed countries are affected by this condition during their first year of life. While the incidence tends to decrease as children grow older, the onset of symptoms can occur at any age^13,14^. CMP, particularly casein and whey proteins, are commonly used as excipients in pharmaceutical formulations^15^. These proteins can serve various purposes in drug formulations, such as stabilizing agents, emulsifiers, or carriers^16,17^. Although the incidence of reactions to cow’s milk allergens in medications among sensitized patients has not been thoroughly studied, it seems to be low but increasing. Nonetheless, the potential for severe reactions warrants careful consideration when administering medications that might contain milk allergens to such children^17^. Additionally, inter-lot variability in DPIs shows clinically significant and seemingly random differences in milk protein contamination^18^. Therefore, patients with CMPA need to make every effort to avoid common food and non-food products containing the problematic proteins.

Confusion between LI and CMPA is common, primarily due to their shared source, milk, and overlapping symptoms, including diarrhea, colic, and other gastrointestinal, respiratory, and skin issues^15^. LI is a digestive issue caused by insufficient activity of the enzyme lactase. It can be primary (rarer and more severe) or more often congenital, making it necessary to exclude or eat only small quantities of products containing lactose (depending on the degree of intolerance)^19^. In contrast, CMPA results from an immunological response to CMP (caseins and whey proteins: alpha and beta lactalbumins, serum albumin, immunoglobulin, and lactoferrin)^20^. The two main subtypes of CMPA are IgE- and non-IgE-mediated allergies, although a mixed presentation caused by the activation of both immunological pathways also exists^14^. IgE-mediated sensitivity to milk proteins is commonly found in individuals who are lactose intolerant^21^.

Treatment focuses predominantly on dietary restriction of dairy products. Management of CMPA involves strict avoidance of CMP, including scrutiny of medication labels for hidden milk protein sources. Dietary modification and lactase enzyme supplementation are commonly recommended strategies to alleviate the symptoms triggered by lactose-containing medications and foods^22^.

These conditions present an ongoing challenge for healthcare providers, as they must carefully evaluate the risk–benefit balance of prescribing lactose-containing medications to patients with LI. This represents a significant socioeconomic burden and impacts the quality of life of affected individuals and their families.

Nevertheless, to the best of the authors knowledge, the assessments of dairy excipients’ presence in drug products are scarce, and those that exist have small sample sizes, underscoring the present survey’s relevance.

This study aimed to examine the prevalence of lactose and other dairy-derived ingredients used as excipients in pharmaceuticals authorized for marketing in Portugal.

## Materials and methods

A search was conducted using the Summary of Product Characteristics for human medications authorized for marketing in Portugal to identify dairy-derived excipients in pharmaceutical products. These SmPCs were accessed through the online database INFOMED (https://extranet.infarmed.pt/INFOMED-fo/). The pharmacotherapeutic groups were selected based on their therapeutic indications, focusing on conditions with high incidence rates in adults and children. The groups included antiasthmatics, non-steroidal anti-inflammatory drugs (NSAIDs), analgesics, and antipyretic drugs. The inclusion criteria comprised medicines from specific groups, such as analgesics and antipyretics containing paracetamol, NSAIDs based on ibuprofen (alone or in combination), and antiasthmatics/bronchodilators. All had to have marketing authorization in Portugal and an accompanying SmPC available on INFOMED. Generic and branded medications were included across all dosages and formulations except injectables. This encompassed both prescription-only and over-the-counter medicines and pediatric and adult formulations.

The presence of lactose, CMP or similar substances was determined by examining the complete list of excipients provided in each SmPC (refer to Table 1).

**Table 1:** Dairy-derived excipients investigated within the Summary of Product Characteristics (SmPC).

Casein	Lactulose
Caseinate	Lactose
Immunoglobulins	Milk compound, milk blend
Lactalbumin	Fermented lactic acid starter culture in milk or whey
Lactitol	Serum Albumin
Lactoferrin	Whey

In addition to the excipients listed in Table 1, several drugs mention flavors and essences (such as cream) in their SmPCs. Since there is no conclusive proof that these compounds are entirely safe for individuals with CMPA or LI, this study assumed, as a precautionary measure, that these excipients could contain dairy derivatives. Medications were categorized as either 'milk allergens present' (MAP) or 'milk allergens free' (MAF) based on the presence of components listed in Table 1, as verified in the SmPC. However, it is essential to note that even if no milk allergens are mentioned in the SmPC, cross-contamination during production cannot be entirely ruled out without explicit assurance from the manufacturing laboratory. Therefore, the classification of medications as MAF in this study is based solely on the composition information provided in the SmPC.

Data analysis was performed using SPSS Statistics version 29.0 for Windows (IBM Corp. Armonk, NY). The statistical significance level was determined as a two-tailed p < 0.05.

## Results and discussion

In this study, 397 drugs were analyzed, including 142 analgesics and antipyretics containing paracetamol, 100 NSAIDs containing ibuprofen and 155 antiasthmatic and bronchodilator medications. The findings are summarized in Table 2.

**Table 2:** Presence of dairy excipients in the studied therapeutic groups according to dosage form.

	N	MAP
**ANALGESIC AND ANTIPYRETICS**	**142**	**7 (4.9%)**
**Solid oral dosage forms**	**101**	**4 (3.9%)**
Capsules	1	0
Coated tablet	4	0
Film-coated tablets	9	0
Tablets	69	0
Granules	1	0
Orodispersible tablets	3	0
Effervescent tablet	10	4
Orodispersible granules	1	0
Effervescent powder	2	0
Powder for oral solution in sachet	1	0
**Liquid oral dosage forms**	**20**	**3 (15%)**
Oral suspension	1	0
Syrup	8	3
Oral solution	11	0
**Rectal dosage forms**	**21**	**0**
Suppository	21	0
**NSAIDs**	**100**	**39 (39%)**
**Solid oral dosage forms**	**78**	**39 (50%)**
Soft capsule	6	0
Coated tablet	4	2
Prolonged release tablet	1	0
Dispersible tablet	1	0
Film-coated tablet	52	36
Effervescent granules	4	1
Granules for oral solution	9	0
Powder for oral suspension	1	0
**Liquid oral dosage forms**	**20**	**0**
Oral suspension	20	0
**Rectal dosage forms**	**2**	**0**
Suppository	2	0
**ANTIASTHMATIC**	**155**	**97 (62.6%)**
**Inhalation dosage forms**	**155**	**97 (62.6%)**
Dry powder for inhalation	74	71
Powder for inhalation, capsule	26	26
Solution for inhalation	3	0
Solution for inhalation by nebulization	6	0
Solution for inhalation by vaporization	1	0
Pressurized solution for inhalation	15	0
Suspension for nebulizer inhalation	4	0
Pressurized suspension for inhalation	26	0

Of the 397 medications reviewed, 143 (36%) were identified as MAP. The primary allergen was lactose, except in three analgesics and antipyretics, where the excipient was cream essence. However, because of the risk of cross-contamination, lactose may contain traces of CMP^18^. Reports of lactose contamination with CMP are rare and usually accidental, but they can lead to adverse reactions, posing significant risks.

The highest prevalence of MAP was found in antiasthmatic drugs with a rate of 62.6%, followed by NSAIDs at 39%. The relationship between pharmacotherapeutic groups and the presence of lactose was examined using the Chi-squared Pearson exact test, which revealed a strong and significant association (*p* < 0.001).

**Figure 1:**
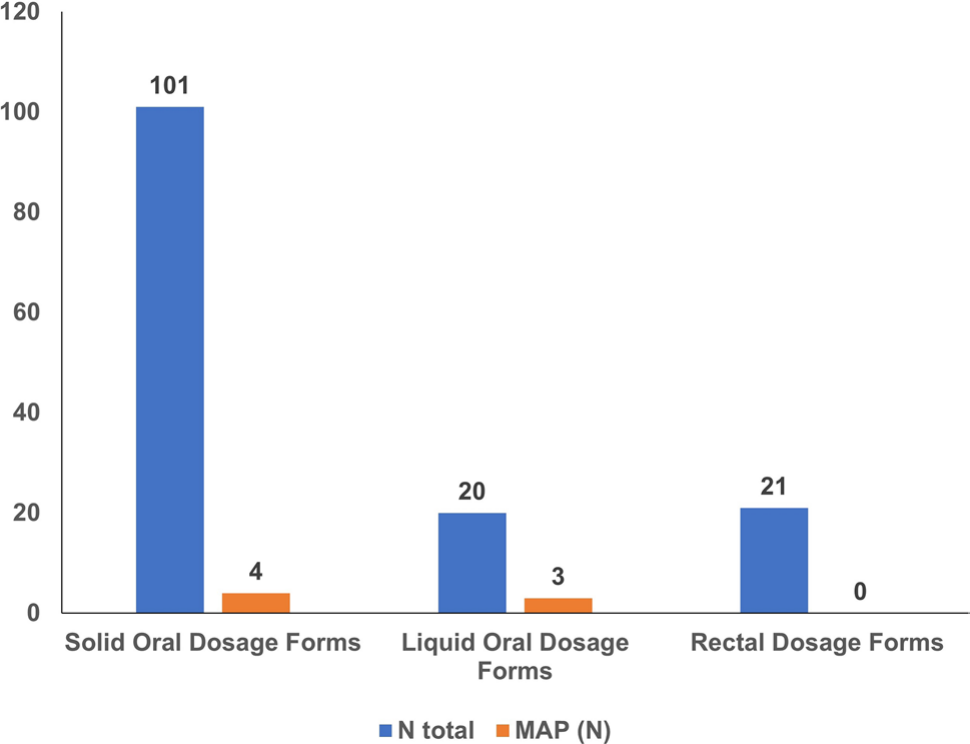
Milk allergens (N) in analgesic and antipyretic drugs by dosage form.

**Figure 2:**
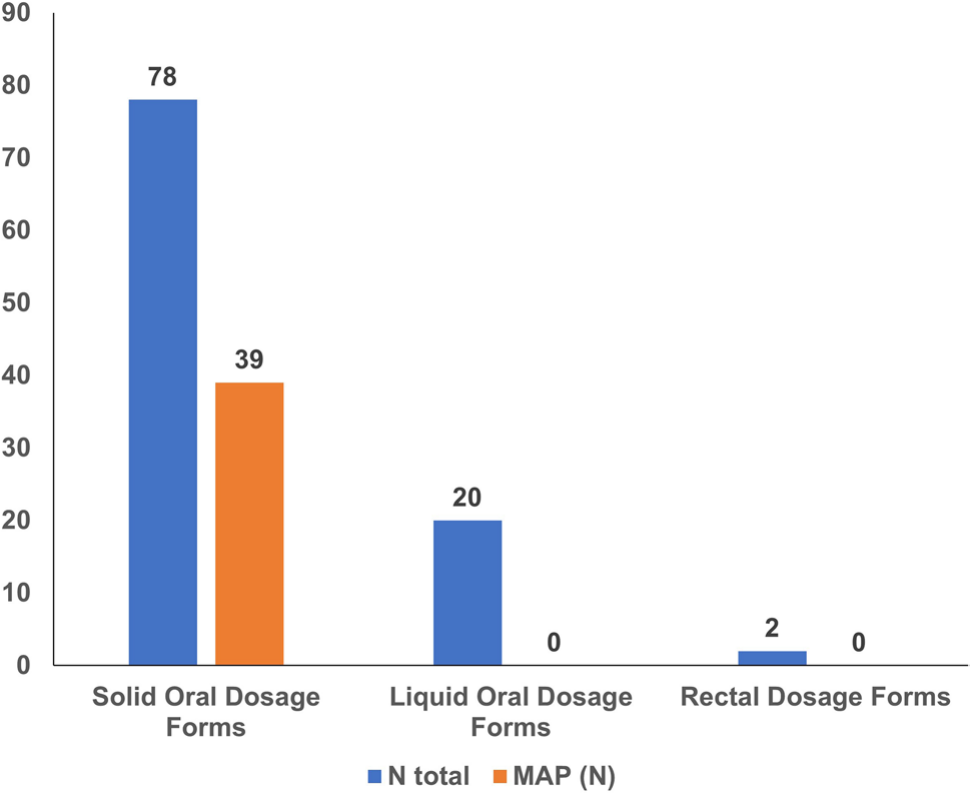
Milk allergens (N) in NSAIDs by dosage form.

**Figure 3:**
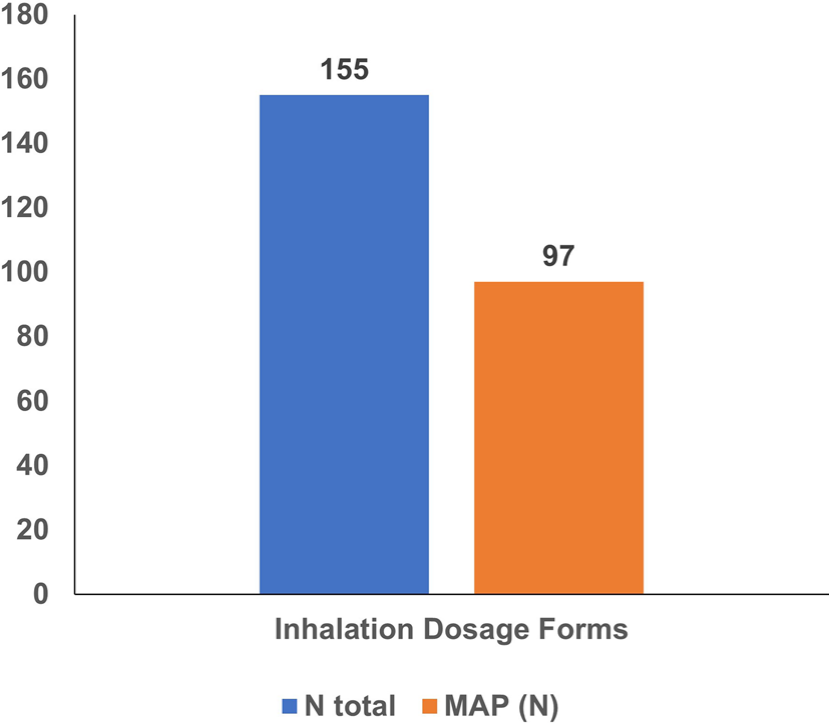
Milk allergens (N) in antiasthmatic drugs.

Regarding pharmaceutical forms, dairy-derived excipients were found in 97% of antiasthmatic powders for inhalation and 69.2% of film-coated tablet NSAIDs (see Table 2). Figures 1, 2, and 3 illustrate the prevalence of medicines containing milk allergen in each therapeutic group studied, based on drug dosage forms such as solid oral, liquid oral, rectal, or inhaled.

Our finding that lactose is predominantly present in the pharmacotherapeutic group of antiasthmatics, particularly in dry powder inhaler (DPIs) formulations, aligns with Santoro et al. (2019), who identified dry powder inhalers as the formulation with the highest prevalence of lactose as an excipient.

The large lactose carrier particles in DPIs often get retained in the oropharynx and are likely swallowed, posing a risk of intolerance in sensitive individuals^6^. Literature indicates that DPIs have the highest number of hypersensitivity reports, likely due to lactose deposition in the oropharynx, occurring in about 98% of cases, leading to ingestion^23^.

In Brazil, a study analyzed the excipients in 181 drugs that induced allergic reactions across several pharmacotherapeutic groups, including NSAIDs, antibacterials, bronchodilators and anticonvulsants. Among these drugs, 28% contained lactose, as identified in the package insert, primarily in capsule and dry powder form and within the group of antiasthmatics and bronchodilators. These results are consistent with the data obtained in our study^24^.

**Figure 4:**
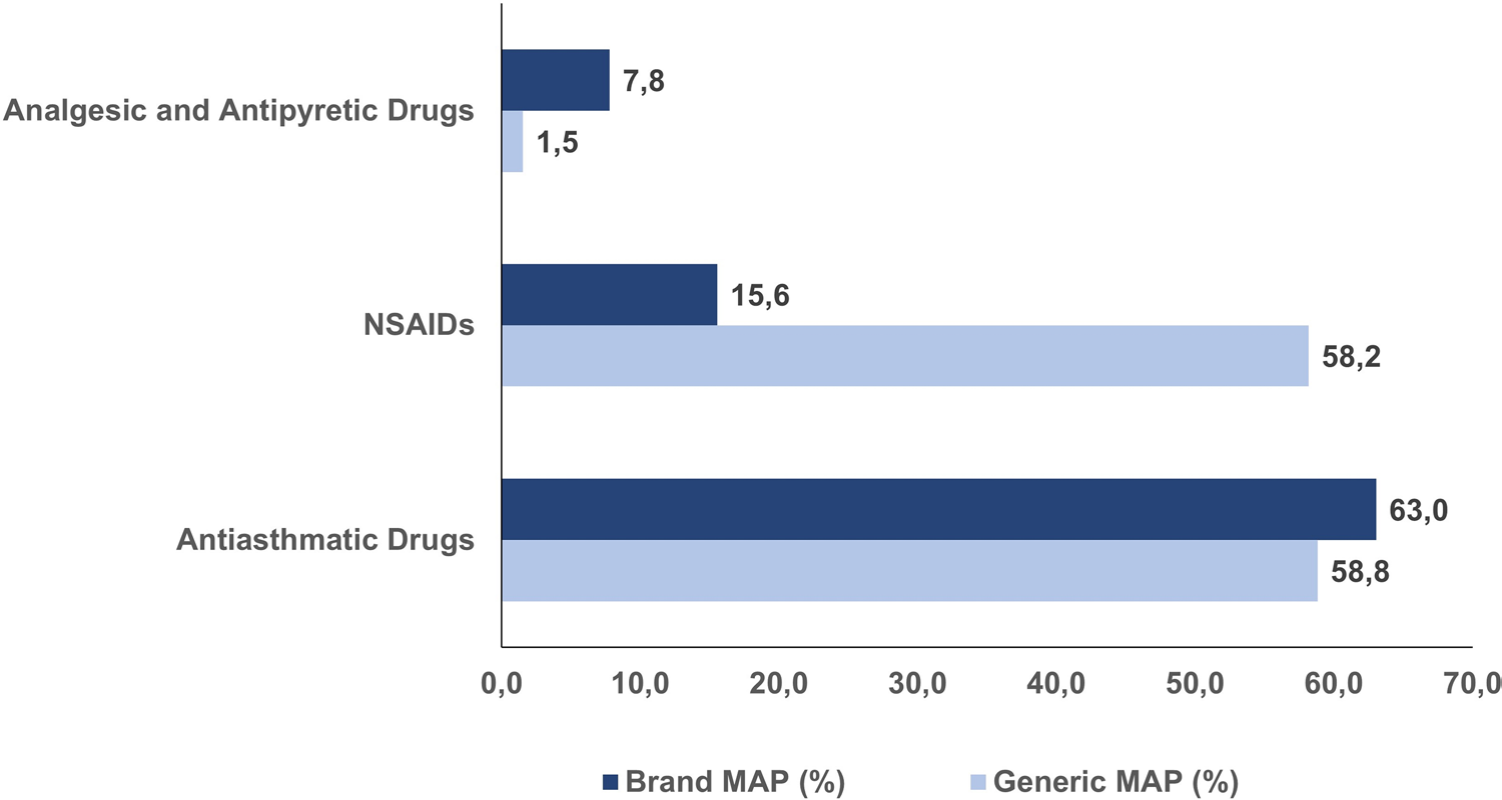
Prevalence (%) of MAP within therapeutic groups, categorized by the type of drug (generic versus branded).

Figure 4 shows the analysis of milk excipient prevalence within each therapeutic group, considering whether the drugs were generic or branded.

Fisher’s exact test was applied to explore the association between the presence of lactose and the classification of drugs as generic or branded, irrespective of therapeutic group. The analysis revealed no statistically significant association (*p* > 0.05). However, when the analysis was conducted considering the therapeutic groups, a statistically significant association was observed for NSAIDs (*p* < 0.001).

The SmPCs do not provide information about the quantity of excipients in medications, leading to prescribers and pharmacists often being unaware of the exact content of dairy derivatives^6^. Additionally, several factors complicate predicting the real impact of lactose and CMP on sensitive individuals: actual lactose intake varies with pH, rate of gastric emptying, and intestinal motility^6^, and sensitivity to lactose varies widely, resulting in differing severities of symptoms^1^. Hence, the frequency of use of lactose does not provide any information in terms of actual degree of exposure, and therefore risk posed to individuals with LI or CMPA. However, the presence of dairy-derived excipients in medicines should always be considered a potential risk for LI individuals, especially those who are highly sensitive.

Our study underscores the importance of health professionals being aware of these risks and assists them in selecting safer alternatives when prescribing and dispensing medications.

## Conclusions

This study revealed that lactose is predominantly used in solid forms of medication, suggesting that liquid or rectal forms may be safer options for patients with severe milk allergies or intolerance.

A significant number of NSAIDs and antiasthmatic drugs contain dairy-derived excipients.

The inclusion of lactose in medications is generally safe for most lactose-intolerant patients due to the minimal amounts used. Since our study focused on qualitative analysis, we could not determine the exact amount of excipients in the analyzed medications. Nevertheless, caution is essential for several reasons: the uncertain lactose content in many medications, highly variable and unpredictable lactose intake, patients' adherence to multi-dose therapeutic regimens, and the unpredictable effects of lactose consumption. Therefore, as a precautionary measure, it is advisable to carefully consider when prescribing medications containing dairy-derived ingredients to patients with LI or CMPA.

Moreover, health professionals must closely monitor lactose-intolerant or CMPA patients to prevent non-adherence issues arising from the fear of symptoms, which could potentially compromise treatment regimens.

Although the SmPCs of the studied medications did not mention any CMP, the presence of lactose can pose a serious risk for individuals with CMPA due to possible allergic reactions. Further research is needed to ascertain the safety of lactose in medicines and its implications for patients with CMPA and LI.

Given the potential impact of lactose on the health of lactose-intolerant individuals and those with CMPA, alongside its widespread use as an excipient in medicines, the pharmaceutical industry is urged to explore suitable alternatives for producing lactose-free medicines.

In summary, thoughtful evaluation, exploring alternative formulations, and diligent monitoring are crucial to minimize these risks and uphold patient safety.

## Data Availability

All data generated or analyzed during this study are included in this published article.
